# Randomized Controlled Trial Evidence on Peroxisome Proliferator-Activated Receptor (PPAR) Agonists in Primary Biliary Cholangitis: A Systematic Review and Meta-Analysis

**DOI:** 10.1155/ijh/8870546

**Published:** 2025-12-02

**Authors:** Marrium Sultan Dar, Tooba Fatima, Syeda Dua Azhar, Zuhaa Rehman, Muhammad Affan, Yusra Muhammad Saleem, Saqib Ali, Fabiha Athar, Samra Ishaq, Noor ul Ain, Jawad Zahid, Aimen Waqar Khan, Haziq Ovais, Tagwa Kalool Fadlalla Ahmad, Khabab Abbasher Hussien Mohamed Ahmed, Hareesha Rishab Bharadwaj

**Affiliations:** ^1^Department of Medicine, Jinnah Sindh Medical University, Karachi, Pakistan; ^2^Department of Medicine, Ahfad University for Women, Omdurman, Sudan; ^3^Faculty of Medicine, University of Khartoum, Khartoum, Sudan; ^4^Faculty of Biology, Medicine and Health, The University of Manchester, Manchester, UK

**Keywords:** meta-analysis, peroxisome proliferator activated receptors agonists, PPAR agonists, primary biliary cholangitis

## Abstract

**Purpose:**

Primary biliary cholangitis (PBC), an autoimmune liver disease, has the potential to advance to liver cirrhosis and result in fatality. Ursodeoxycholic acid (UDCA) is the first-line treatment, while obeticholic acid (OCA) serves as a second-line option because of moderate UDCA nonresponsiveness and cirrhosis-related concerns. Additional therapies are necessary because of recent warnings regarding OCA usage in patients with cirrhosis. This study aimed to evaluate the efficacy and safety of peroxisome proliferator-activated receptor (PPAR) agonists in PBC.

**Methods:**

We searched PubMed, Google Scholar, and the Cochrane Library until October 2023. We included all randomized controlled trials (RCTs) that studied the efficacy and safety of PPAR agonists in treating PBC. The primary outcome of interest was change in alkaline phosphatase (ALP) levels. In contrast, the secondary outcomes were changes in gamma-glutamyl transferase (GGT), alanine transaminase (ALT), aspartate aminotransferase (AST), total bilirubin (TBil), triglyceride levels, and pruritis. We used a random-effects model to calculate the risk ratio (RR) and standardized mean difference (SMD) with 95% CI.

**Results:**

A total of eight RCTs (*n* = 515) were eligible for the analysis. Pooled data showed beneficial effects of PPAR agonists compared with placebo for change in ALP level (SMD = −2.81, 95%CI = −4.10 to − 1.51; *p* < 0.0001, I^2^ = 96%), GGT level (SMD = −1.29, 95%CI = −2.09 to − 0.48; *p* = 0.002, I^2^ = 92%), TBil level (SMD = −0.77, 95%CI = −1.32 to − 0.22; *p* = 0.006, I^2^ = 86%), and Tg level (SMD = −0.99, 95%CI = −1.63 to − 0.35; *p* = 0.003, I^2^ = 83%). There was no significant difference between PPAR agonists and placebo for ALT level (SMD = −0.93, 95%CI = −1.94 to 0.08; *p* = 0.07, I^2^ = 95%), AST level (SMD = −0.01, 95%CI = −0.67 to 0.66; *p* = 0.99, I^2^ = 91%), and pruritus (RR = 0.77, 95%CI = 0.29 to 2.06; *p* = 0.60, I^2^ = 34%).

**Conclusion:**

Our study found a superior efficacy of PPAR agonists compared with placebo for change in ALP, GGT, TBil, and Tg levels, highlighting the potentially beneficial effect of PPAR agonists on liver health.

## 1. Introduction

Primary biliary cholangitis (PBC) is a chronic, progressive autoimmune cholangitic hepatic disease [1–7]. It is primarily a disease of middle-aged women, affecting approximately 20–40 per 100,000 people globally. It is suspected initially by the persistent elevation of serum alkaline phosphatase (ALP) and later associated with raised gamma-glutamyl transferase (GGT), aminotransferase activity, and total bilirubin (TBil) [2–5, 8]. Though the precise cause is unknown, the immune-mediated inflammation and scarring of bile ducts lead to bile buildup, further exacerbating the hepatocyte damage [6, 8]. PBC, when left untreated, can lead to cirrhosis [1–3], hepatocellular carcinoma (HCC) [6], hepatic failure, fibrosis, the need for a transplant, and even death [1, 2, 4, 7, 8]. The goal of therapy is to slow down the progression, normalize the liver function test, and prevent or treat complications that may arise while enhancing the quality of life of the patients [5, 8].

At present, there is no cure for PBC; however, two drugs, ursodeoxycholic acid (UDCA) and obeticholic acid (OCA), have been approved as standard therapy [1–8]. UDCA, a non-cytotoxic bile acid, has been a therapeutic mainstay for decades. It decreases the biochemical markers of cholestasis, ALP, and GGT while also extending transplant-free survival [1–4, 6]. However, in the long run, up to 40% of patients had inadequate biochemical responses to UDCA, which translates into deteriorated long-term survival [1–5]. Hence, OCA, a selective agonist of the farnesoid X receptor (FXR), has been introduced as an add-on therapy that acts by improving biochemical response by increasing bile acid secretion [1–5, 8]. However, despite its superior efficacy over UDCA, it is associated with inducing dose-dependent severe pruritus or worsening it [1–3, 5, 8] and cannot be given to patients with advanced cirrhosis [9].

Peroxisome proliferator-activated receptor (PPAR) agonists are nuclear receptor agonists that act by modulating gene expression and are increasingly recognized as promising therapeutic agents [1–3, 5]. They modulate various cellular processes and are anti-inflammatory [2, 4, 5]. PPARs exist in three subtypes: alpha (*α*), gamma (*γ*), and delta (*δ*) [2, 5]. PPAR-*α* is predominantly expressed in the liver, along with in skeletal muscle and the heart [5]. Fenofibrate, a selective PPAR-*α* agonist, improves liver function tests, decreases ALP levels, and enhances lipid profiles, though the safety profile remains controversial [2, 3, 5]. PPAR-*δ* is ubiquitously expressed in various tissues, like Kupffer cells, cholangiocytes, and hepatic stellate cells [2, 5]. Seladelpar, a potent and selective PPAR-*δ* agonist, has an anti-inflammatory and antifibrotic effect and also lowers levels of biochemical markers of cholestasis in patients with PBC [2, 5, 8]. PPAR-*γ*, though universally present, is mainly found in adipose tissue and immune cells. Saroglitazar, a novel dual PPAR agonist with potent and predominant PPAR-*α* activity and moderate PPAR-*γ* agonistic activity, improves insulin resistance and lipid profile [5]. Another dual PPAR agonist is elafibranor, a PPAR-*α*/*δ* agonist [4, 5]. It is antifibrotic and enhances the liver function profile [4]. Bezafibrate, a pan-PPAR agonist, modulates anti-inflammatory effects and bile acid metabolism via multiple pathways and normalizes cholestasis biomarkers (ALP, GGT, and TBil), further improving transplant-free survival [1, 3–7].

The recent clinical trials proved PPAR agonist to be efficacious in comparison with UDCA, OCA, and other drugs in patients with UDCA-refractory PBC. This meta-analysis aimed to evaluate the efficacy and safety of different PPAR agonists in PBC. The findings of this particular study will guide clinicians in the selection of a specific PPAR agonist as an add-on therapy for managing patients with PBC.

## 2. Methods

### 2.1. Data Sources and Search Strategy

Our systematic review and meta-analysis are reported using the Preferred Reporting Items for Systematic Reviews and Meta-Analysis statement (PRISMA) [10] and are registered at PROSPERO under the ID: CRD42024446911. PubMed, Cochrane, and Google Scholar were searched thoroughly from inception through October 2023. No restrictions were placed on the year of the publication. Two independent investigators (MSD and MA) conducted a thorough systematic review using a search strategy as outlined in the Supporting Information, Table S1.

### 2.2. Study Selection

All the articles were screened by two independent investigators (MA and MSD). The studies were first screened based on the title and abstract and then further evaluated based on the full text. We only selected randomized controlled trials (RCTs) for our analysis and excluded reviews, case reports, case series, observational studies, editorials, commentaries, and preclinical studies. Only articles in the English language were included. The inclusion criteria were set based on author discussion and consensus. There was no restriction on the size of the studies considered for inclusion.

### 2.3. Data Extraction and Outcomes of Interest

To ensure comprehensive data extraction, a structured data collection form was employed through an Excel Sheet to extract relevant information from each study. Two authors (MSD and ZA) independently extracted data from the selected studies. The baseline characteristics that were extracted included: (1) The first author's last name, year of publication, sample size, and intervention period; (2) the primary outcome of interest for this meta-analysis, which was change in ALP; and (3) the secondary outcomes, which were changes in GGT, ALT, AST, TBil, total triglyceride, and incidence of pruritis. The quality of RCTs was assessed using the revised Cochrane risk-of-bias tool for randomized trials (RoB 2.0) [11].

### 2.4. Statistical Analyses

Standardized mean difference (SMD) was calculated for continuous outcomes, and risk ratios (RRs) were used for dichotomous outcomes. Heterogeneity was assessed using Higgins I^2^ statistic [12]. Given the heterogeneity in study designs and patient populations, a random-effect model was used for the primary analysis [13]. Publication bias was not assessed as the number of studies included was < 10 [14]. For this study, statistical significance was set at 5%. Review Manager (Version 5.3; Cochrane Collaboration, Oxford, United Kingdom) was used for all other analyses.

### 2.5. Study Selection

**Figure 1:**
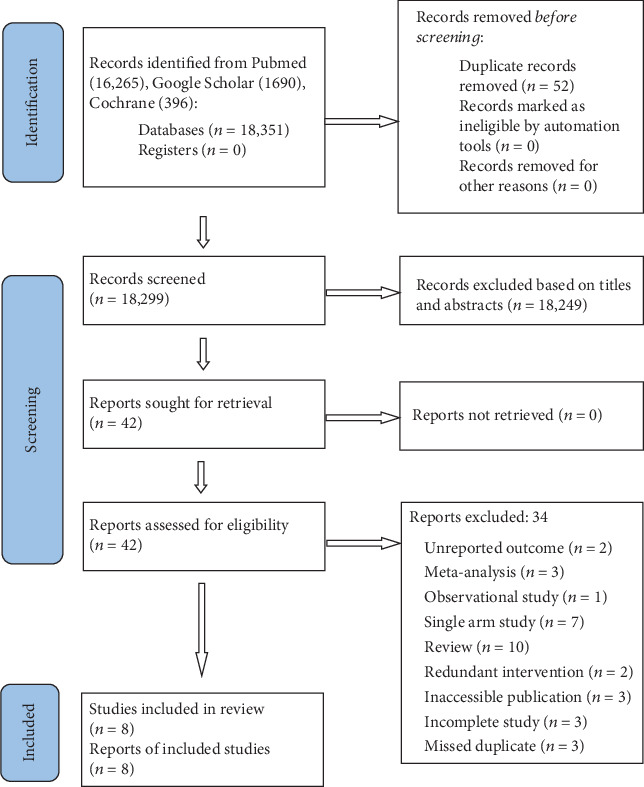
PRISMA flowchart.

Of the initial 18,351 articles generated from the database search, 52 were eliminated because of duplication. Subsequently, 18,299 records underwent screening based on their initial identification, excluding 18,249 records because of inadequate relevance (studies involving non-PBC populations or articles identified as reviews) found in their titles and abstracts. Following this screening phase, 42 reports were identified for retrieval. Of these, only eight reports [1–8] met the criteria based on full text and were included in the final meta-analysis, and all were RCTs.  Figure 1 describes this meta-analysis's Preferred Reporting Items for Systematic Reviews and Meta-Analyses (PRISMA).

### 2.6. Baseline Characteristics

The baseline characteristics of the included RCTs are shown in Table 1. Eight eligible RCTs [1–8] were included in the present meta-analysis, including 515 patients. The publication years of the involved articles varied from 2004 to 2023. In the meta-analysis, all eight RCTs included in the study utilized interventions involving PPAR agonists as the treatment group and placebo as the comparator group. The detailed characteristics of included RCTs are presented in the Supporting Information (Table S2).

### 2.7. Quality Assessment and Publication Bias

The Cochrane method of evaluating RCTs indicated that of the eight RCTs included in the meta-analysis, six studies were deemed at high risk of bias [1–4, 7, 8]. Two studies had a high risk of bias because of open-label nature [6, 7]. Two studies had biases because of the involvement of the funding source in data analysis [2, 8] (Figure S1).

### 2.8. Primary Outcome

#### 2.8.1. Effect on ALP Levels

**Figure 2:**
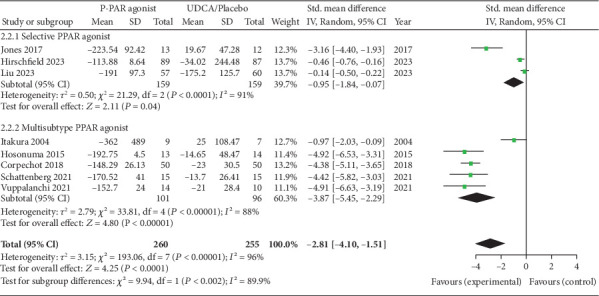
Forest plot of change in ALP levels FROM BASELINE.

All the studies reported changes in serum ALP [1–8]. There was a significant difference between PPAR agonists and the placebo group in reducing ALP levels (SMD = −2.81; 95%CI = −4.10 to − 1.51; *p* = 0.0001; I^2^ = 96%). On performing subgroup analysis, both selective PPAR agonists (SMD = −0.95; 95%CI = −1.84 to − 0.07; *p* = 0.04) and multi-PPAR agonists (SMD = −3.87; 95%CI = −5.45 to − 2.29; *p* = 0.0001) showed a significant decrease in ALP levels compared with placebo. However, a significant heterogeneity persisted between the groups (Figure 2).

### 2.9. Secondary Outcomes

#### 2.9.1. Effect on GGT Levels

Six studies reported changes in GGT levels [1, 3–6, 8]. There was a statistically significant difference between PPAR agonists and placebo in reducing GGT levels (−1.29; 95%CI = −2.09 to − 0.48; I^2^ = 92%). Subgroup analysis revealed that both selective PPAR agonists (SMD = −0.40; 95%CI = −0.63 to − 0.17; *p* = 0.0008) and multi-PPAR agonists (SMD = −1.83; 95%CI = −2.80 to − 0.86; *p* = 0.0002) showed a significant decrease in GGT levels compared with placebo. Both subgroups had significant heterogeneity. The figure of the forest plot is given in the Supporting Information (Figure S2).

#### 2.9.2. Effect on ALT Levels

Changes in ALT levels were reported by six studies [1, 3–6, 8]. There was no statistically significant difference between PPAR agonists and placebo treatment groups in reducing ALT levels (SMD = −0.93; 95%CI = −1.94 to 0.08; *p* = 0.07; I^2^ = 95%). Subgroup analysis was performed, which revealed a nonsignificant difference between the selective PPAR agonist group and multiselective PPAR agonist group in reducing ALT levels compared with placebo with significant heterogeneity in both subgroups. The figure of the forest plot is given in the Supporting Information (Figure S3).

#### 2.9.3. Effect on AST Levels

Changes in AST levels were reported by seven studies [1, 3–8]. There was no statistically significant difference between PPAR agonists and placebo treatment groups in reducing AST levels (SMD = −0.01; 95%CI = −0.67 to 0.66; *p* = 0.99; I^2^ = 95%). Subgroup analysis was performed, which revealed a nonsignificant difference between the selective PPAR agonist group and multi-PPAR agonist group in reducing AST levels compared with placebo with significant heterogeneity in both subgroups. The figure of the forest plot is given in the Supporting Information (Figure S4).

#### 2.9.4. Effect on TBil Levels

Seven studies reported changes in TBil levels [1, 3–8]. There was a statistically significant difference between PPAR agonists and placebo in reducing TBil levels (SMD = −0.77; 95%CI = −1.32 to − 0.22; *p* = 0.06; I^2^ = 86%). Subgroup analysis showed that TBil levels were reduced in both the selective PPAR agonists group (SMD = −0.25; CI = −0.49 to − 0.02; *p* = 0.04) and multi-PPAR agonists group (SMD = −1.03; CI = −1.76 to − 0.30; *p* = 0.006) compared with placebo. Heterogeneity became negligible in the selective PPAR agonists group while it remained significant in the multi-PPAR agonists group. The figure of the forest plot is given in the Supporting Information (Figure S5).

#### 2.9.5. Effect on Triglyceride Levels

Five studies reported changes in triglyceride levels [3–6, 8]. There was a significant difference between PPAR agonists and placebo treatment groups in reducing triglyceride levels (SMD = −0.99; 95%CI = −1.63 to − 0.35; *p* = 0.003; I^2^ = 83%). Upon performing subgroup analysis, there was no significant difference found between selective PPAR agonists and placebo while multi-PPAR agonists were more efficacious in reducing triglyceride levels (SMD = −1; 95%CI = −1.51 to − 0.49; *p* = 0.0001). Both subgroups had significant heterogeneity. The figure of the forest plot is given in the Supporting Information (Figure S6).

#### 2.9.6. Effect on Pruritus

Only five studies reported pruritus [1–4, 8]. There was no significant difference between PPAR agonists and placebo with regard to causing pruritus (RR = 0.77; 95%CI = 0.29 to 2.06; *p* = 0.19; I^2^ = 34%). The figure of the forest plot is given in the Supporting Information (Figure S7).

## 3. Discussion

PBC is a rare autoimmune liver disease predominantly affecting women. It is characterized by the progressive inflammation and destruction of small bile ducts in the liver, leading to cholestasis, which causes severe fatigue and itching. If left untreated, PBC can advance to cirrhosis, end-stage liver disease, and even be fatal [15]. The primary objective of medical intervention is the amelioration of distressing symptoms such as pruritus and fatigue to improve the quality of life, the normalization of liver function tests, and the deceleration of disease progression and a paramount objective is the prevention or postponement of liver fibrosis and cirrhosis development. UDCA stands as the primary therapeutic regimen for managing PBC, though it is notable that a significant segment, potentially up to 40%, of patients fails to achieve the desired response according to specific criteria [16, 17]. This subset of nonresponders exhibits considerably poorer transplant-free survival rates in contrast to their UDCA-responsive counterparts. OCA, initially FDA-approved for patients with PBC who do not respond to or cannot tolerate UDCA, is now subject to certain limitations, particularly when dealing with advanced cirrhosis. Notably, OCA is contraindicated in cases of decompensated cirrhosis and compensated cirrhosis with indications of portal hypertension [18]. Moreover, it is important to recognize that OCA itself may be accompanied by low tolerability and a propensity to induce pruritus as an adverse effect. The dearth of FDA-approved therapies for PBC underscores the compelling necessity for the exploration of alternative treatment modalities.

Clinical investigations in the form of RCTs have been carried out to assess the effectiveness and safety of PPAR agonists for the treatment of PBC. There exist three distinct isotypes of PPARs—PPAR-*α*, PPAR-*γ*, and PPAR-*β*/*δ*—each encoded by separate genes and characterized by specific tissue distribution and functions [19]. These agents can help in PBC by their anti-inflammatory, metabolic regulation, fibrosis modulation, and immunomodulation. They are categorized into selective and multi-PPAR. Selective PPAR agonists include fenofibrate, known for its affinity to PPAR-*α*, and seladelpar, which primarily activates PPAR-*δ*. Multi-PPAR agonists encompass compounds such as bezafibrate (considered a pan-PPAR agonist), saroglitazar (dual PPAR-*α* and PPAR-*γ* agonist), and elafibranor (dual PPAR-*α* and PPAR-*δ*).

We have done a pooled analysis of eight RCTs to evaluate the efficacy and safety of PPAR agonists in PBC. The primary outcome of interest was ALP while the secondary outcomes were GGT, ALT, AST, TBil, triglyceride, and pruritis. Pooled data showed beneficial effects of PPAR agonists compared with placebo for reduction in ALP, GGT, TBil, and triglycerides levels. There was no significant difference between PPAR agonists and placebo for ALT, AST, and pruritus.

Meta-analysis of pooled data has demonstrated a statistically significant reduction in ALP levels when comparing PPAR agonists with a placebo group. These findings are consistent with a previous meta-analysis, which showed that using bezafibrate, a PPAR agonist, in combination with UDCA results in significant improvement in serum ALP levels [20]. To minimize the heterogeneity within the dataset, we conducted subgroup analyses by categorizing PPAR agonists into two main groups: selective PPAR agonists and multi-PPAR agonists. The subgroup analysis revealed that both selective PPAR agonists and multi-agonists led to a significant reduction in ALP levels when compared with the placebo group. However, it is noteworthy that despite this subgroup division, heterogeneity remained relatively high. This observed heterogeneity may be attributed to several factors. Firstly, variability in the types of PPAR agonists within the subgroups contributes to the heterogeneity. For instance, some studies, such as Liu et al., exclusively employed selective PPAR agonists like fenofibrate (an alpha agonist), while others, like Hirschfield, utilized seladelpar (a delta agonist). Meanwhile, the remaining studies employed multisubtype PPAR agonists. This divergence in PPAR agonist selection introduces a source of heterogeneity due to distinct mechanisms of action and potential variations in treatment outcomes. Secondly, differences in drug dosage and the duration of treatment regimens among the included studies may also account for the persisting heterogeneity. Variations in dosing and the duration of exposure to PPAR agonists could influence treatment response and result in differential effects on ALP levels. Further, the selection, performance, detection and biases due to the conflicts of interest in some studies could be another reason for this significant heterogeneity. Our analysis revealed a statistically significant reduction in GGT levels associated with the use of PPAR agonists. The subgroup analysis, stratified according to the type of PPAR agonists, demonstrated a significant reduction in GGT levels within both the selective and multi-PPAR agonist subgroups when compared with placebo. The persistent heterogeneity observed in both subgroups can be attributed to several factors, which include specific types of PPAR agonists used, treatment duration, and biases across studies as mentioned earlier. PPAR activators have been shown to inhibit the activation of inflammatory response genes, including cytokines such as IL-2, IL-6, IL-8, and TNF-alpha, as well as metalloproteases. These inflammatory mediators are often elevated in conditions associated with liver injury and can contribute to the pathogenesis of liver diseases. By suppressing the production of proinflammatory cytokines and metalloproteases, PPAR activators help mitigate inflammation in the liver, thereby reducing damage and ALP release [21].

Our analysis of ALT levels did not reveal any significant difference between the PPAR agonist group and the placebo group (*p* = 0.07, I^2^ = 95%). Similarly, for AST levels, there was no significant difference observed between the PPAR agonist and placebo groups (*p* = 0.99, I^2^ = 91%). For further assessment of both outcomes, subgroup analysis was conducted; however, it did not affect the heterogeneity of the overall outcome. This variation may be attributed to several factors, one of which could be that some studies recruited participants undergoing treatment with UDCA for several months, while others had inadequate responses to conventional treatment before participating in the clinical trials. Furthermore, some participants, such as those included in the study by Liu et al., had never received UDCA for PBC before participating in their clinical trial. These differences may have affected baseline aminotransferase levels and subsequently influenced the treatment response. In a previous meta-analysis conducted by Yin et al. on bezafibrate, the most extensively investigated PPAR agonist for PBC, a significant reduction in ALT levels (MD = −10.24 IU/L, *p* = 0.00001) [22] was reported. While our pooled analysis did not reveal statistically significant reductions in either aminotransferase levels, an intriguing observation emerged. The study that exhibited the most significant reduction in ALT (MD = −3.00) and AST (MD = −1.74) levels, included in our meta-analysis, was conducted by Corpechot et al. in 2018 [1] with a trial duration of 24 months. In contrast, most of the studies in our meta-analysis had trial durations ranging from 12 to 52 weeks. Therefore, longer treatment durations and more extensive multicentered controlled studies may be necessary in the future to fully understand the effects of PPAR agonists on aminotransferase levels. The proposed mechanism for reducing aminotransferases is believed to result from the anti-inflammatory action of PPAR agonists. This mechanism involves the upregulation of anti-inflammatory genes, particularly interleukin-1 receptor antagonist (IL-1ra) and inhibitor of nuclear factor kappa B alpha (I*κ*B*α*), which act as inhibitors of NF*κ*B found in hepatocytes (PPAR-*α*, PPAR-*δ*) and Kupffer cells (PPAR-*δ*) in the liver .

Our results demonstrated a noteworthy decrease in TBil levels (SMD = −0.77, *p* = 0.006, I^2^ = 86%). Subsequently, we conducted subgroup analysis, and within the selective PPAR agonist subgroup, a remarkable reduction in heterogeneity was achieved, reaching 0%. At the same time, it remained high for the multigroup, which may be attributed to the fact that multiple drugs were used for treatment that target different PPAR receptor subtypes. While all studies included in our analysis demonstrated a reduction in TBil levels, Hosonuma et al. [7] and Corpechot et al. [1], in their clinical trial with bezafibrate (selective for PPAR-*α* receptor), reported the most significant reduction. The potential mechanism for reducing TBil levels by PPAR agonists can be explained in four distinct ways. First and foremost, PPAR-*α* has been shown to downregulate the expression of *CYP27A1* and *CYP7A1*, which are pivotal enzymes involved in bile acid synthesis. Simultaneously, it upregulates the expression of *BSEP* and *MRP2* genes, which are crucial in bile acid secretion. Furthermore, it mitigates bile acid toxicity by activating the multidrug-resistant gene, which yields a protein (*ABCB4*) that integrates into the canalicular membrane of liver cells, facilitating bile acid secretion, particularly biliary phosphatidylcholine. Finally, it contributes to the detoxification of bile acids by upregulating the expression of critical enzymes such as CYP3A4, UGT, SULT2A1, and others [23]. Our analysis findings align with the outcomes of several trials investigating the efficacy of both fibrates and nonfibrates in reducing bilirubin levels. For instance, in a 2021 clinical trial by Soret et al. involving fibrates (specifically, bezafibrate and fenofibrate), administered as part of a triple regimen therapy (UCDA, OCA, and either fibrate) for 11 months, a significant reduction in bilirubin levels was reported. However, some experts recommend cautious use of fenofibrate in patients with cirrhosis because of specific trials reporting a rapid increase in bilirubin levels [22]. Moreover, a multicentric observational study conducted in United Kingdom, which included 457 patients, did not reveal any significant differences in biochemical response when comparing fibrates with OCA [24].

Regarding triglyceride levels, there was an overall significant reduction observed when PPAR agonists were compared with the placebo (MD = −0.99, *p* = 0.003, I^2^ = 83%), except for one study conducted by Itakura et al. in 2004 [6], which reported a slight increase in triglyceride levels. Despite the significant reduction, the high overall heterogeneity of 83% prompted us to conduct the subgroup analysis. Similar to the results of our analysis, Yin et al., in their meta-analysis evaluating bezafibrate, concluded an overall significant reduction in triglyceride level (MD, −26.84 mg/dL, *p* = 0.0001) [22]. Moreover, another meta-analysis evaluating fenofibrate found a significant reduction in triglyceride levels that included six trials with 84 patients with PBC (MD: −0.41 mg/dL, *p* = 0.05) [25]. The observed reduction in triglyceride levels may be attributed to mechanisms elucidated through experiments conducted on mice models studying different PPAR receptor subtypes. PPAR-*α* is primarily expressed in tissues responsible for fatty acid oxidation, including the liver, heart, kidney, skeletal muscle, and adipose tissue. In hepatocytes, it plays a pivotal role in transcriptionally regulating genes associated with beta-oxidation, glucose metabolism, bile acid regulation, and lipid transport. The activation of PPAR-*α* in hepatocytes increases beta-oxidation, leading to a reduction in plasma triglyceride levels and an elevation in high-density lipoprotein levels [22]. Conversely, PPAR*β*/*δ* predominantly governs beta-oxidation in peripheral organs, whereas PPAR-*γ* is primarily responsible for storing triglycerides in adipose tissue [26].

Pruritis remains the most common symptom of PBC. It can be due to bile acids, which results in the activation of G protein-coupled receptor pathways [27, 28]. Also, a lysophosphatidic acid-producing enzyme, autotaxin, is thought to cause pruritis in PBC [29]. Interleukin-31 (IL-31) produced by Type 2 helper T-cells is also thought to cause pruritis [30]. UDCA has no role in the improvement of pruritis while OCA worsens it. We analyzed to assess the safety of PPAR agonists in terms of pruritus. The overall analysis revealed no significant difference between intervention and control groups in causing pruritus (*p* = 0.6). However, studies by Jones et al. [2] and Hirschfield et al. [8], both having seladelpar as an intervention group, found that more patients suffered from pruritis in the intervention group compared with the control group while Corpechot et al. [1] and Liu et al. [3], having bezafibrate and fenofibrate as intervention, observed a noteworthy decrease in the prevalence of pruritus among individuals receiving the intervention compared with those in the control group. The study by Schattenberg et al. [4], using elafibranor as an intervention, found no significant difference between intervention and control groups in causing pruritis. Nonetheless, it raised patients' baseline visual analogue scale (VAS) and PBC 40 quality of life scores who reported pruritus. Some pilot studies and case series have been conducted, which demonstrated the beneficial effects of bezafibrate in preventing pruritis [31, 32]. Similarly, previous studies have demonstrated the beneficial effects of seladelpar and elafibranor in reducing and not worsening pruritis. PPAR agonists are thought to reduce IL-31 and bile acid levels thereby improving pruritis [33, 34].

Overall, it is noteworthy that groups treated with multi-PPAR agonists demonstrated greater mean improvements and more pronounced reductions in biochemical markers compared with those receiving selective PPAR agonists, underscoring the potential advantage of multiagents in the management of PBC.

To the best of our knowledge, it is the first meta-analysis that explored the PPAR agonists (alpha, gamma, and delta) and checked its safety in terms of pruritus and efficacy in terms of serum ALP, GGT, ALT, AST, TBil, and Tg levels. Comprehensive databases were searched, applying techniques such as snowballing, ensuring that no relevant articles were left out. However, our study has some limitations. Our study had high heterogeneity for which subgroup analysis was conducted based on the type of PPAR agonists. Heterogeneity persisted across the subgroups for all outcomes except for TBil where it was reduced to negligible in the selective PPAR agonists group. Subgroup analyses based on factors such as age, prior treatments, drug choice, treatment duration, and dosage were not feasible in our meta-analysis because the included studies were inherently heterogeneous in design and did not report stratified outcomes. For example, one trial [3] enrolled only treatment-naïve patients while others [1, 2, 4–8] focused solely on UDCA inadequate responders; treatment durations were fixed and varied widely between studies, and each trial evaluated a single drug or predefined doses without sufficient within-study variability. Moreover, the small sample sizes in early-phase trials precluded meaningful stratification. These structural differences, rather than analytic limitations, contributed to the observed heterogeneity, and our forest plots ensure the individual study contributions remain transparent. Our RCTs have relatively small sample sizes, which are not adequate to represent the entire population and yield significant results. Also, treatment durations were different ranging from 12 weeks to 8 years, which can be found in Supplementary Table S2. Also, studies by Hosonuma et al. and Liu et al. had data reported in median and interquartile ranges representing skewness across these trials. Not all the RCTs reported all the outcomes hence few studies with small sizes might fail to address the actual efficacy and safety of drugs. Despite encountering these limitations, we put our efforts into yielding meaningful and impactful results, which would further help in advancing knowledge.

## 4. Conclusion

Our systematic review and meta-analysis suggests that PPAR agonists exhibit superior efficacy as compared with placebo in reducing key biochemical markers in PBC including ALP, GGT, TBil, and triglyceride levels, which indicates beneficial effects on liver function and overall metabolic profile. However, the analysis did not find significant differences for ALT, AST, and pruritus suggesting that while PPAR agonists effectively target certain liver enzymes and markers, their impact on other aspects of liver function and patient symptoms may be limited. Further research is necessary to fully understand the therapeutic potential and optimize the clinical use of PPAR agonists in managing PBC.

## Figures and Tables

**Table 1:** Baseline characteristics of included RCTs.

**Study**	**Intervention type and dosage**	**ALP (IU/L)**		**GGT (IU/L)**		**ALT (IU/L)**		**TBil (mg/dL)**		**Triglycerides (mg/dL)**		**AST (IU/L)**	
		**Control group**	**Intervention group**	**Control group**	**Intervention group**	**Control group**	**Intervention group**	**Control group**	**Intervention group**	**Control group**	**Intervention group**	**Control group**	**Intervention group**
Itakura et al. 2004^a^ [6]	Bezafibrate (400 mg/day)	436 (40)	618 (183)	130 (43)	171 (57)	50 (9)	55 (111)	—	—	105 (33)	113 (20)	42 (9)	60 (15)
Hosonuma et al. 2015^b^ [7]	Bezafibrate (400 mg/day)	454 (355–792)	423 (359–798)	52 (16–252)	92 (23–368)	25 (12–74)	26 (15–78)	0.7 (0.2–1.5)	0.7 (0.3–2.1)	117 (58–279)	105 (40–308)	27 (17–74)	31 (16–75)
Jones et al. 2017^c^ [2]	Seladelpar (50 mg)	233 (73)	312 (95)	183 (123)	220 (152)	40 (24)	47 (31)	0·68 (0·35)	0·73 (0·27)			36 (12)	37 (18)
Corpechot et al. 2018 [1]	Bezafibrate (400 mg/day)	242 (186–344)^b^	244 (211–308)^b^	164 (100–273)^b^	162 (112–240)^b^	53 (34–72)^b^	55 (37–73)^b^	⁣^∗^12.6 ± 6.8^a^	⁣^∗^14.0 ± 7.6^a^	—	—	45 (33–64)^b^	44 (33–57)^b^
Schattenberg et al. 2021^c^ [4]	Elafibranor (80 mg)	296.2 (115.5)	350.6 (152.1)	229.6 (115.9)	282.3 (215.7)	48.5 (22.3)	57.6 (24.7)	0.651 (0.259)	0.577 (0.389)	—	—	46.7 (15.8)	54.3 (18.7)
Vuppalanchi et al. 2021^c^ [5]	Saroglitazar (2 mg)	294.9 (73.3)	351.3 (161.3)	188.6 (111.2)	383.0 (499.6)	44.4 (24.1)	56.5 (34.8)	0.7 (0.2)	0.6 (0.2)	112.4 (61.2)	112.5 (68.9)	37.1 (14.6)	46.6 (21.1)
Hirschfield et al. 2023^c^ [8]	Seladelpar (5 mg)	293.4 (106.2)	290.5 (104.2)	228.9 (193.0)	231.3 (212.0)	44.4 (20.7)	47.7 (21.0)	0.71 (0.32)	0.76 (0.35)	—	—	37.5 (16.8)	40.1 (14.5)
Liu et al. 2023^b^ [3]	Fenofibrate (400 mg)	303 (218–503)	277 (202–426)	327 (201–476)	343 (176–591)	63 (38–98)	54 (40–77)	⁣^∗^18.4 (11.7–26.0)	⁣^∗^15.7 (11.5–25.6)	1.1 (0.8–1.5)	1.2 (0.8–1.6)	66 (46–93)	59 (43–95)

Abbreviations: ALP, alkaline phosphatase; ALT, alanine aminotransferase; GGT, gamma-glutamyl transferase; TBil, total bilirubin.

^a^Data reported in mean (SE).

^b^Data expressed as median (IQR).

^**c**^Data reported in mean (SD).

⁣^∗^TBil reported in *μ*mol/L.

## Data Availability

All data generated or analyzed during this study are included in this published article and its Supporting Information files.

## Supporting Information

Supporting Information 1 Supplementary Figure S1. Risk of bias graph and summary. Supplementary Figure S2. Forest plot of change in GGT levels from baseline. Supplementary Figure S3. Forest plot of change in ALT levels from baseline. Supplementary Figure S4. Forest plot of change in AST levels from baseline. Supplementary Figure S5. Forest plot of change in total bilirubin levels from baseline. Supplementary Figure S6. Forest plot of change in triglycerides levels from baseline. Supplementary Figure S7. Forest plot of pruritus as an adverse effect of the drug.

Supporting Information 2 Supplementary Table S1. Detailed search strategy. Supplementary Table S2. Characteristics of RCTs.
