# Supplementary material for: Randomized Controlled Trial Evidence on Peroxisome Proliferator-Activated Receptor (PPAR) Agonists in Primary Biliary Cholangitis: A Systematic Review and Meta-Analysis
Source: Int J Hepatol. 2025 Dec 2;2025:8870546. doi: 10.1155/ijh/8870546 (PMC12688640; doi:10.1155/ijh/8870546)
Supplement: Supporting Information 1 — Supplementary Figure S1. Risk of bias graph and summary. Supplementary Figure S2. Forest plot of change in GGT levels from baseline. Supplementary Figure S3. Forest plot of change in ALT levels from baseline. Supplementary Figure S4. Forest plot of change in AST levels from baseline. Supplementary Figure S5. Forest plot of change in total bilirubin levels from baseline. Supplementary Figure S6. Forest plot of change in triglycerides levels from baseline. Supplementary Figure S7. Forest plot of pruritus as an adverse effect of the drug. [file 8870546.f1.docx]

**Title: Efficacy and Safety of Peroxisome Proliferator-activated Receptor Agonists in Primary Biliary Cholangitis:** **A Systematic Review and Meta-Analysis of Randomized Controlled Trials**


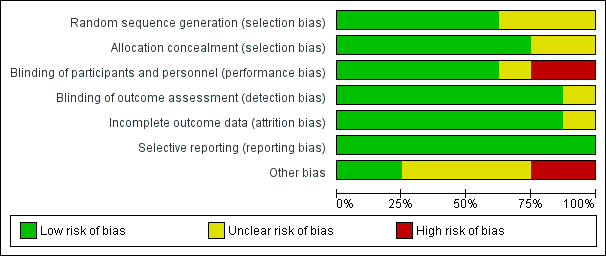


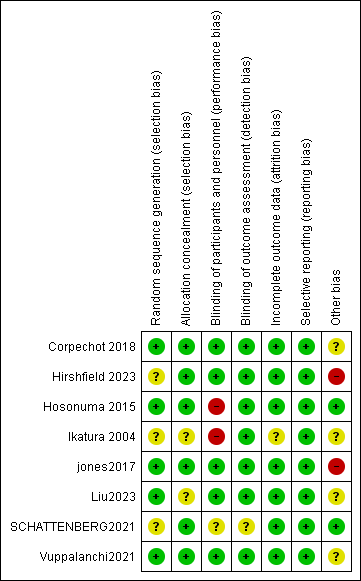


***Supplementary Figure S1.*** Risk of Bias Graph and Summary


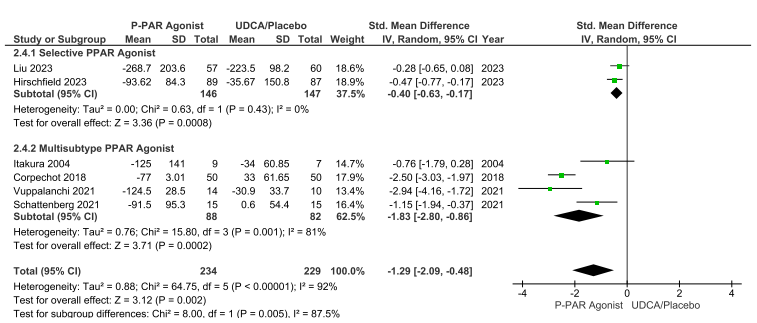


***Supplementary Figure S2.*** Forest Plot of Change in GGT Levels from Baseline


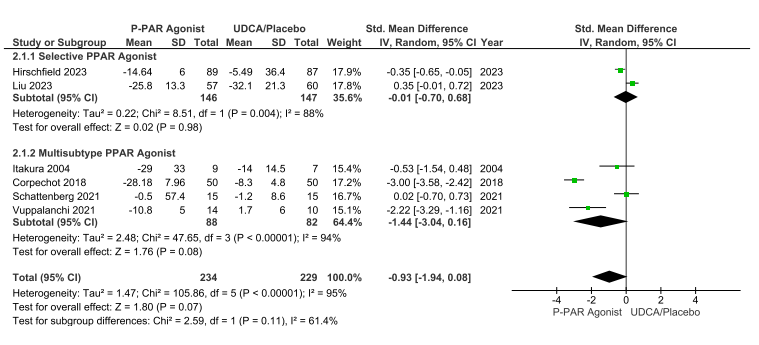


***Supplementary Figure S3.*** Forest Plot of Change in ALT Levels from Baseline


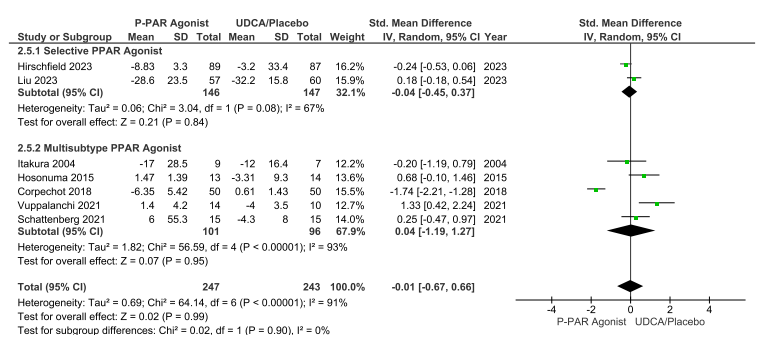


***Supplementary Figure S4.*** Forest Plot of Change in AST Levels from Baseline


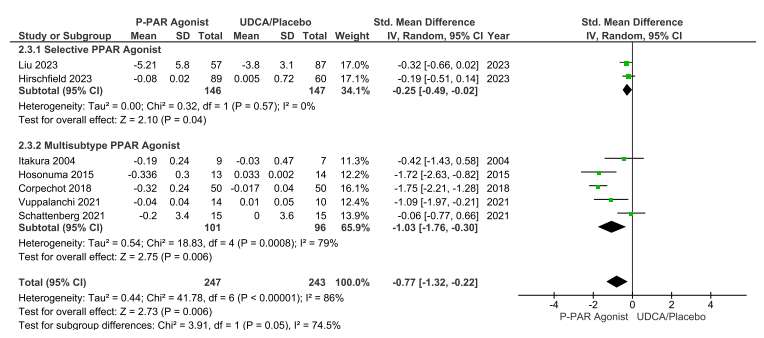


***Supplementary Figure S5.*** Forest Plot of Change in Total Bilirubin Levels from Baseline


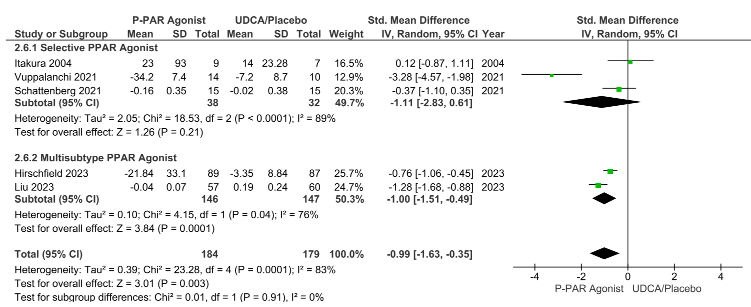


***Supplementary Figure S6.*** Forest Plot of Change in Triglyerides Levels from Baseline


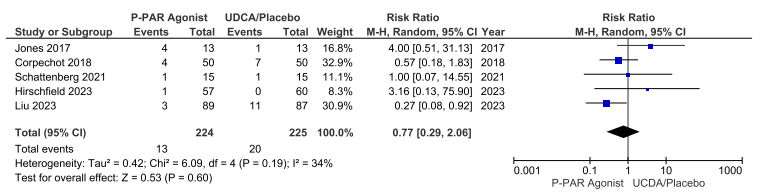


***Supplementary Figure S7.*** Forest Plot of Pruritus as an adverse effect of the drug
