# Supplementary material for: Randomized Controlled Trial Evidence on Peroxisome Proliferator-Activated Receptor (PPAR) Agonists in Primary Biliary Cholangitis: A Systematic Review and Meta-Analysis
Source: Int J Hepatol. 2025 Dec 2;2025:8870546. doi: 10.1155/ijh/8870546 (PMC12688640; doi:10.1155/ijh/8870546)
Supplement: Supporting Information 2 — Supplementary Table S1. Detailed search strategy. Supplementary Table S2. Characteristics of RCTs. [file 8870546.f2.docx]

**Title: Efficacy and Safety of Peroxisome Proliferator-activated Receptor Agonists in Primary Biliary Cholangitis:** **A Systematic Review and Meta-Analysis of Randomized Controlled Trials**

*Supplementary Table S1.* Detailed search strategy

| **Database** | **Search Strategy** | **Results** |
| --- | --- | --- |
| PubMed | (((((((((((("liver cirrhosis, biliary"[MeSH Terms]) OR (Primary Biliary Cholangitis)) OR (Primary Biliary Cirrhosis)) OR (PBC)) OR (Chronic Non-suppurative Cholangitis)) OR (Autoimmune Cholangitis))) AND (PPAR agonists) OR (PPAR alpha agonists) OR (PPAR gamma Agonists)) OR (PPAR delta agonists)) OR (PPAR ligands)) OR (PPAR modulators) | 15,965 |
| Google Scholar | "Primary Biliary Cholangitis"\|"Primary Biliary Cirrhosis"\|"Chronic Non-suppurative Cholangitis"\|"Autoimmune Cholangitis"\|"PBC" "PPAR agonists"\|"Peroxisome Proliferator-Activated Receptor agonists"\|"PPAR alpha agonists"\|"PPAR gamma” | 1690 |
| Cochrane | "Primary Biliary Cholangitis" in Title Abstract Keyword OR "primary biliary cirrhosis" in Title Abstract Keyword AND "PPAR agonists" in Title Abstract Keyword OR "Peroxisome Proliferator-Activated Receptor agonists" in Title Abstract Keyword OR "PPAR ligands" in Title Abstract Keyword | 396 |

*Supplementary Table S2.* Characteristics of RCTs

| Character-istics | Itakura  2004 | Hosonuma  2015 | Jones  2017 | Corpechot 2018 | Schattenberg 2021 | Vuppalanchi 2021 | Hirshfield 2023 | liu 2023 |
| --- | --- | --- | --- | --- | --- | --- | --- | --- |
|  |  |  |  |  |  |  |  |  |
| Study name | Prospective randomized crossover trial of combination therapy with bezafibrate and UDCA for primary biliary cirrhossis | A Prospective Randomized Controlled Study of  Long-Term Combination Therapy Using Ursodeoxycholic  Acid and Bezafi brate in Patients With Primary Biliary  Cirrhosis and Dyslipidemia | Seladelpar (MBX-8025), a selective PPAR-δ agonist, in patients  with primary biliary cholangitis with an inadequate response  to ursodeoxycholic acid: a double-blind, randomised,  placebo-controlled, phase 2, proof-of-concept study | A Placebo-Controlled Trial of Bezafibrate  in Primary Biliary Cholangitis | A randomized placebo-controlled trial of elafibranor in patients  with primary biliary cholangitis and incomplete response to UDCA | Proof-of-concept study to evaluate the safety and efficacy of saroglitazar in patients with primary biliary  cholangitis | Seladelpar efficacy and safety at 3 months in patients with  primary biliary cholangitis: ENHANCE, a phase 3,  randomized, placebo-controlled study | Effectiveness of fenofibrate in treatment-naïve patients with primary biliary cholangitis A Randomized Clinical Trial |
| Patients, n | 9 | 27 | 41 | 100 | 45 | 37 | 265 | 117 |
| Enrollment initiation | 2001 | 2003 | 2015 | 2012 | 2017 | 2017 | 2018 | 2016 |
| Enrollment completion | 2002 | 2005 | 2016 | 2014 | 2018 | 2020 | 2019 | 2021 |
| Year of completion |  |  |  |  | 2018 |  | 2020 | 2022 |
| Population | patients who were diagnosed with PBC at Musashino Red Cross Hospital between March 2001 and june 2002 |  | Study included patients aged 18–75 years who recieve diagnosis of primary biliary cholangitis at 29 centres in Europe and North America. The diagnosis required the presence of at least two of the following criteria: a history of alkaline phosphatase above the upper limit of normal (ULN) for at least 6 months, a positive autoantibody test (anti-mitochondrial antibody >1:40 on immunofluorescence or M2 positivity by enzyme-linked immune sorbent assay or positive anti-nuclear antibodies specific to primary biliary cholangitis), and a documented liver biopsy consistent with primary biliary cholangitis. Patients were required to be on a stable and recommended dose of ursodeoxycholic acid for the past 12 months and to have an alkaline phosphatase of at least 1·67 times the ULN. | Patient 18 years of age diagnosed with primary biliary cholangitis according to provided criteria were enrolled at 21 centers of France. At the time of recruitment all the patients were being treated with Ursodeoxycholic acid at a dose of 13 to 15 mg/kg/day. Only those patients were considered eligible who had not significant biochemical response to Ursodexoycholic acid defined accrding to established Paris 2 criteria ( i.e serum level of alkaline phosphatase or aspartate aminotransferase >1.5 times the upper limit of the normal range or an abnormal total bilirubin level) after 6 months or more of treatment. | This study included patients aged 18 to 75 years with PBC as shown by the presence of at least 2 of the following 3 diagnostic factors: i) a history of elevated ALP levels for at least 6 months prior to randomization, ii) liver biopsy consistent with PBC, iii) positive anti-mitochondrial antibody titre (>1/40 on immunofluorescence or M2 positive by ELISA) or positive PBC specific anti-nuclear antibodies, All patients were treated with UDCA for at least 12 months and were at a stable dose for at least 6 months prior to randomization. At inclusion, patients were required to have ALP levels >−1.67x ULN (ULN = 104 U/L for females; 129 U/L for males). | Patients with 18 to 75 years of age who had a diagnosis of PBC based  on theEuropean Association for the Study of Liver and American Association for the Study of Liver Disease . The entry criteria was inadequate response to a year of  ursodiol therapy and ALP level of at least 1.67 X ULN range at both screening  visits 1 and 2 with <30% variance and total bilirubin less than or equal to 2 X ULN. | Patients aged 18 to 75 years who were diagnosed with PBC [≥ 2 of the following criteria: history of ALP > ULN for ≥ 6 months, positive anti-mitochondrial antibody titers (> 1/40 on immunofluorescence or M2-positive by ELISA) or PBC-specific antinuclear antibodies, or histology liver biopsy consistent with PBC] were screened for recruitment. Patients with ALP ≥ 1.67×ULN and total bilirubin ≤2×ULN were included Patients must have been receiving a stable and recommended UDCA dose (generally 13–15 mg/kg/d) for the prior 12 months unless they were UDCA intolerant. | Patients who were diagnosed with PBC were allocated from Xijing Hospital based on the presence of two of the following three criteria: presence of anti-mitochondrial antibody or other PBC-associated autoantibodies, including sp100 or gp210; elevation of ALP; compatible or diagnostic liver histology. All patients were recruited at initial diagnosis and had never received Ursodexoycholic acid before. |
| Trial type | Randomized controlled trial | prospective, multicenter, randomized trial | Multicentre,double blind,randomise, placebo-controlled, parallel trial | two-group, ran-  domized, double-blind, placebo-controlled trial. |  |  | double-blind, randomized, placebo-  controlled study | randomized, parallel and open-label clinical trial. |
| Inclusion  criteria | Sixteen patients were included in the present study, 12  women and four men with PBC who were diagnosed at the  Musashino Red Cross Hospital between March 2001 and Their diagnoses were confirmed by serum bio-  chemical tests, which showed in each case at least a 1.3-fold  elevated alkaline phosphatase level, concomitant with at  least a 40-fold positive excess of anti-mitochondrial antibod-  ies, as determined by the reversed passive hem-agglutination  tests. |  | Study included patients aged 18–75 years who recieve diagnosis of primary biliary cholangitis at 29 centres in Europe and North America. The diagnosis required the presence of at least two of the following criteria: a history of alkaline phosphatase above the upper limit of normal (ULN) for at least 6 months, a positive autoantibody test (anti-mitochondrial antibody >1:40 on immunofluorescence or M2 positivity by enzyme-linked immune sorbent assay or positive anti-nuclear antibodies specific to primary biliary cholangitis), and a documented liver biopsy consistent with primary biliary cholangitis. Patients were required to be on a stable and recommended dose of ursodeoxycholic acid for the past 12 months and to have an alkaline phosphatase of at least 1·67 times the ULN. | Patients 18 years of age or older were diagnosed of primary biliary cholangitis according to provided criteria were enrolled at 21 centers of France. At the time of recruitment all the patients were being treated with Ursodeoxycholic acid at a dose of 13 to 15 mg/kg/day. Only those patients were considered eligible who had not significant biochemical response to Ursodexoycholic acid defined accrding to established Paris 2 criteria ( i.e serum level of alkaline phosphatase or aspartate aminotransferase >1.5 times the upper limit of the normal range or an abnormal total bilirubin level) after 6 months or more of treatment. | This study included patients aged 18 to 75 years with PBC as shown by the presence of at least 2 of the following 3 diagnostic factors: i) a history of elevated ALP levels for at least 6 months prior to randomization, ii) liver biopsy consistent with PBC, iii) positive anti-mitochondrial antibody titre (>1/40 on immunofluorescence or M2 positive by ELISA) or positive PBC specific anti-nuclear antibodies, All patients were treated with UDCA for at least 12 months and were at a stable dose for at least 6 months prior to randomization. At inclusion, patients were required to have ALP levels >−1.67x ULN (ULN = 104 U/L for females; 129 U/L for males). | Patients with 18 to 75 years of age who had a diagnosis of PBC based  on theEuropean Association for the Study of Liver and American Association for the Study of Liver Disease . The entry criteria was inadequate response to a year of  ursodiol therapy and ALP level of at least 1.67 X ULN range at both screening  visits 1 and 2 with <30% variance and total bilirubin less than or equal to 2 X ULN. | .  Patients aged 18 to 75 years who were diagnosed with PBC [≥ 2 of the following criteria: history of ALP > ULN for ≥ 6 months, positive anti-mitochondrial antibody titers (> 1/40 on immunofluorescence or M2-positive by ELISA) or PBC-specific antinuclear antibodies, or histology liver biopsy consistent with PBC] were screened for recruitment. Patients with ALP ≥ 1.67×ULN and total bilirubin ≤2×ULN were included Patients must have been receiving a stable and recommended UDCA dose (generally 13–15 mg/kg/d) for the prior 12 months unless they were UDCA intolerant. | Patients who were diagnosed with PBC were allocated from Xijing Hospital based on the presence of two of the following three criteria: presence of anti-mitochondrial antibody or other PBC-associated autoantibodies, including sp100 or gp210; elevation of ALP; compatible or diagnostic liver histology. All patients were recruited at initial diagnosis and had never received Ursodexoycholic acid before. |
| Exclusion criteria | Patients  were excluded from this study if histologically overlapping  with autoimmune hepatitis, if positive of serum antigen or  antibody associated with the hepatitis B virus, and if pos-  itive of serum antibody of hepatitis C virus or human im-  munodeficiency virus. Patients with esophageal varices in  which endoscopical treatment was necessary, with ascites,  with a history of drinking excessive amounts of alcohol or  drug use, or with cardiac or renal failure, or with hepatocel-  lular carcinoma, were also excluded. |  | Patients were excluded if they had any other liver disease, or any medical condition that would confound the results, or compromise  their safety. Other exclusions were alanine aminotransferase (ALT) or aspartate aminotransferase (AST) concentrationsmore than three times the ULN, total bilirubin more thantwo times the ULN, and creatine kinase or serum creatinine above the ULN. The use of colchicine, methotrexate,azathioprine, or systemic steroids within 2 months before  screening was not permitted. Patients taking fibrates or simvastatin were also excluded, as well as any patients experimental primary biliary cholangitis treatment,  including obeticholic acid. | Patients with a total bilirubin level above 50 μmol per liter (3 mg per deciliter) were excluded and Patients with typical features of autoimmune hepatitis were also excluded from the trial. | The main exclusion criteria were i) other liver diseases including viral hepatitis (HBV and HCV), alcohol-related liver disease, primary sclerosing cholangitis (PSC) , autoimmune hepatitis or overlap, history of alpha 1-antitrypsin deficiency, non-alcoholic steatohepatitis (NASH) ii) alanine aminotransferase (ALT) or aspartate aminotransferase (AST)>5xULN, total bilirubin >2xULN, platelet count <150x103, albumin <3.5 g/dl; iii) moderate or severe hepatic impairment (Child-Pugh B/C); iv) current model for end-stage liver disease (MELD) score >−15, signs and symptoms of cirrhosis/portal hypertension including oesophageal varices, ascites, history of variceal bleeding, history of liver transplantation, hepatic encephalopathy current placement on a liver transplant list, history of bacterial peritonitis, hepatocellular carcinoma or hepato-renal syndrome. Excluded medications were defined as follows: fibrates, obeticholic acid and glitazones within 2 months prior toscreening, azathioprine, colchicine, cyclosporine, methotrexate, mycophenolate mofetil, pentoxifylline; budesonide and other systemic corticosteroids within 3 months prior to screening, or immunotherapy directed against interleukins or other cytokines or chemokines within 12 months prior to screening visit. | Key exclusion criteria  included history of liver decompensation with ascites, hepatic encephalopathy, large varices or  concomitant aetiology such as hepatitis B or C virus infection, alcoholic liver disease, definite autoimmune  hepatitis (including patients with overlapping PBC and autoimmune hepatitis) and non-alcoholic  steatohepatitis. | Exclusion  criteria included advanced  PBC as defined by the Rotterdam criteria (coincident  albumin less than the lower limit of normal and total  bilirubin > 1×ULN), aspartate aminotransferase (AST) or  alanine aminotransferase (ALT) >3×ULN,creatinine kinase >1×ULN,  international normalized ratio > 1×ULN, circulating  platelet count of <100×103/μL, esti-  mated glomerular filtration rate <60 mL/min/1.73 m2,clinically significant  hepatic decompensation or presence of another chronic  liver disease, or any other medical condition that would  compromise safety or confound study results. | Patients with any other liver diseases were excluded. |
| Treatment | Patients were allocated randomly into two groups, group  A or group B, and were followed for 1 year (Fig. 1). Patients  assigned to group A received 400 mg/day of bezafibrate and  600 mg/day of UDCA for the first 6 months of study, and for  the remaining 6 months they received 600 mg/day of UDCA  alone. Patients allocated to group B received 600 mg/day of  UDCA for the first half of the study, and 400 mg/day of  bezafibrate and 600 mg/day of UDCA for latter half of the  study. |  |  | Patients were received once daily oral placebo oe benzafibrate at dose of 400 mg, however patients in both groups recieved Ursodexoxycholic acid | Using an Interactive  Response Technology centralized randomization system, in a  1:1:1 ratio to receive elafibranor 80 mg, elafibranor 120 mg, or  placebo once daily for 12 weeks. UDCA treatment was continued  throughout the study and maintained thereafter. |  | Eligible patients were randomly assigned in a 1:1:1 ratio to receive a daily dose of saroglitazar 2  mg, saroglitazar 4 mg, or placebo for 16 weeks in addition to their ongoing treatment with UDCA. | Randomization was computer generated to receive either UDCA alone or a combination of oral fenofibrate at a dose of 200 mg  once-daily and Ursodoxycholic aid at a dose of 13-15 mg/kg/day. |
| Primary outcome |  | Change in baseline serum ALP levels, severity of PBC and transplant-free survival were the main primary end points. Additionally changes in baseline bilirubin, aspartate transaminase and albumin serum levels were also determined | Change in baseline ALP levels over twelve weeks | Normal serum levels of ALP, aspartate aminotransferase, alanine aminotransferase, bilirubin and albumin | Change in baseline ALP levels at week 16 | Reduction in baseline ALP at week 16 | Reduction of or more than 15% in baseline ALP levels at 3 months | Biochemical response percentage in patients in accordance with the Barcelona criterion. Changes in ALP |
| Secondary outcome |  | Safety of the treatment groups. Analysis of the change in serum creatinine, cytokine and interleukin-18 levels | Tolerability, safety and efficacy of treatment. Efficacy was analyzed by serum ALP less than 1.6 times the ULN, normal total bilirubin, ALT, AST, GGT, total cholesterol, HDL, LDL | Percentages of patients with response and normal ALP levels at 24 months, changes AST, ALT, GGT, total bilirubin, total cholesterol, HDL and LDL cholesterol | The percent-  age of patients with a normal ALP level at 24 months; changes in serum levels  of ALP, aspartate aminotrans-  ferase, alanine aminotransferase, γ-glutamyl-  transferase, total bilirubin, albumin, total cho-  lesterol, high-density lipoprotein cholesterol, and  low-density lipoprotein cholesterol, and changes  in the prothrombin index and platelet count; the  percentage of patients with an adequate bio-  chemical response at 24 months | The percentage of patients with improvements in ALP levels, changes in serum AST, ALT, GGT, total bilirubin, total cholesterol, triglycerides, HDL, LDL, serum bile acids, FGF 19 and changes in quality of life | The proportion of patients with normal ALP and change in pruritus NRS at 3 and 6 months. Other secondary endpoints included changes in serum AST, ALT, GGT and lipid levels | The biochemical response percentage, percentage of ALP normalization at different times, changes in serum AST, ALT, GGT, total bilirubin and total cholesterol. changes in liver stiffness, APRI score and FIB-4 score were also included. |
| Intervention Period |  |  |  |  |  |  | Patients were randomized 1:1:1 to oral seladelpar 5 mg  (n=89), 10 mg (n=89), placebo (n=87) daily (with UDCA, as appropriate). |  |
